# Role of Dietary Pattern Analysis in Determining Cognitive Status in Elderly Australian Adults

**DOI:** 10.3390/nu7021052

**Published:** 2015-02-04

**Authors:** Kimberly Ashby-Mitchell, Anna Peeters, Kaarin J. Anstey

**Affiliations:** 1 Centre for Research on Ageing, Health & Wellbeing, The Australian National University, Florey, Building 54, Mills Road, Acton, ACT 2601, Australia; E-Mail: Kaarin.anstey@anu.edu.au; 2 Baker IDI Heart and Diabetes Institute, 75 Commercial Rd, Melbourne VIC 3004, Australia; E-Mail: anna.peeters@bakeridi.edu.au

**Keywords:** dietary pattern, principal component analysis, cognitive impairment, Australia

## Abstract

Principal Component Analysis (PCA) was used to determine the association between dietary patterns and cognitive function and to examine how classification systems based on food groups and food items affect levels of association between diet and cognitive function. The present study focuses on the older segment of the Australian Diabetes, Obesity and Lifestyle Study (AusDiab) sample (age 60+) that completed the food frequency questionnaire at Wave 1 (1999/2000) and the mini-mental state examination and tests of memory, verbal ability and processing speed at Wave 3 (2012). Three methods were used in order to classify these foods before applying PCA. In the first instance, the 101 individual food items asked about in the questionnaire were used (no categorisation). In the second and third instances, foods were combined and reduced to 32 and 20 food groups, respectively, based on nutrient content and culinary usage—a method employed in several other published studies for PCA. Logistic regression analysis and generalized linear modelling was used to analyse the relationship between PCA-derived dietary patterns and cognitive outcome. Broader food group classifications resulted in a greater proportion of food use variance in the sample being explained (use of 101 individual foods explained 23.22% of total food use, while use of 32 and 20 food groups explained 29.74% and 30.74% of total variance in food use in the sample, respectively). Three dietary patterns were found to be associated with decreased odds of cognitive impairment (CI). Dietary patterns derived from 101 individual food items showed that for every one unit increase in ((Fruit and Vegetable Pattern: *p* = 0.030, OR 1.061, confidence interval: 1.006–1.118); (Fish, Legumes and Vegetable Pattern: *p* = 0.040, OR 1.032, confidence interval: 1.001–1.064); (Dairy, Cereal and Eggs Pattern: *p* = 0.003, OR 1.020, confidence interval: 1.007–1.033)), the odds of cognitive impairment decreased. Different results were observed when the effect of dietary patterns on memory, processing speed and vocabulary were examined. Complex patterns of associations between dietary factors and cognition were evident, with the most consistent finding being the protective effects of high vegetable and plant-based food item consumption and negative effects of ‘Western’ patterns on cognition. Further long-term studies and investigation of the best methods for dietary measurement are needed to better understand diet-disease relationships in this age group.

## 1. Introduction

Cognitive impairment is a condition in which a person has difficulty with memory, learning, concentrating or making decisions that affect their daily life [1]. Diet is among several modifiable factors found to influence cognitive function [2,3,4]. Age is presently the strongest known predictor for cognitive decline, and cognitive impairment (CI) has been shown to adversely affect quality of life and functional ability [5,6]. Risk reduction is especially important because there is still no effective treatment for dementia [7].

Studies aimed at elucidating the association between diet and cognitive function have utilised both the single nutrient and dietary pattern approaches [8,9]. While the single-nutrient approach has addressed various public health problems, many researchers theorise that due to high correlations between individual food constituents, there should be a shift toward analysis using a dietary pattern approach [10,11]. Evidence on the effect of dietary lipids, B-vitamins, antioxidants, fish, alcohol, vegetables and legumes have all produced varying results, and further research is needed into biomarkers for particular nutrients and cognitive endpoints in order for any definitive population-based conclusions to be reached [2,12,13,14,15]. Diets low in saturated fat, high in legumes, fruits and vegetables, moderate in ethanol intake and low in meat and dairy have also been highlighted as being beneficial to neurological function. One of the most studied dietary patterns is the Mediterranean diet, a diet rich in cereals, olive oil, fish, fruits and vegetables and low in dairy and meat, with a moderate consumption of red wine. This diet has been linked to increased survival, reduced risk of cancers, cardiovascular disease, longevity and cognitive impairment [16]. However, it is important to consider that there may be other dietary patterns, yet to be identified, that may have similar benefits and that can be applied to various sociocultural and demographic settings.

Few studies have examined the effect of dietary patterns on cognitive function using a data-driven method and even fewer of these studies have utilised Australian data. We identified only two studies utilising a data-driven approach to dietary analysis that have examined links with cognition in an Australian sample. The first used data from the Melbourne Collaborative Cohort Study in conducting factor analysis to determine the effect of dietary intake on psychological distress in older Australians [17] and the second utilised data from the Personality and Total Health (PATH) Through Life Study to examine the diet-depression relationship in three cohorts [18].

PCA is a data-driven approach that reduces a large number of food variables into a smaller set that captures the major dietary traits in the population [19]. In nutritional epidemiology, PCA can be used to investigate exposure-disease associations. As it relates to older age groups, such information can serve to develop age-specific guidelines and policies. One of the major criticisms of PCA, however, is that results can differ based on the methods employed during variable reduction and classification [20,21] and there is presently no accepted gold standard for dietary analysis to guide researchers.

The present study therefore has two aims. First, it addresses the question of how classification systems used to reduce food variables before the application of PCA affect the observed association between diet and cognitive function. Second, it evaluates the association between dietary patterns and cognitive function in a population-based cohort of Australian adults. In addition, it aims to determine the variance in food use explained by the different variable reduction methods employed, *i.e.* using 101 individual food items, 32 food groups and 20 food groups.

## 2. Experimental Section

### 2.1. Study Design and Sample

The study utilised secondary data derived from the AusDiab study, a population-based national survey of the general (non-institutionalised) Australian population aged 25 years and older [22]. The baseline examination was undertaken in 1999–2000 (*n* = 11,247), with follow-ups conducted in 2004–2005 (*n* = 8798) and 2011–2012 (*n* = 6186) [22]. Dietary data were obtained from a sub-group of the sample using a questionnaire (Wave 1: *n* = 3298) [22]. Measurement of cognitive function was conducted on those who attended survey sites in the third Wave of data collection (*n* = 4764) [23]. The present study focuses on the older segment of the sample (age 60+ at baseline) that completed the food frequency questionnaire at Wave 1 and the mini-mental state examination and tests of memory, verbal ability and processing speed at Wave 3 (*n* = 577).

We excluded 2721 participants from the current analysis since these participants had no dietary and/or cognitive data recorded.

### 2.2. Cognitive Outcome Measurement

The mini-mental state examination (MMSE) was used for data collection in 2011–2012 (AusDiab Wave 3) to determine CI status. Participants were classified based on their MMSE score as either cognitively impaired (score of 0–23) or not cognitively impaired (score of 24–30) [24].

The California Verbal Learning Test (CVLT) was used to assess memory using a 16-point scoring system. For this test, participants were asked to recall and repeat a list of 16 common shopping items that had been read to them by an interviewer. During a short delay of 20 min, during which participants were given other tasks to perform, the interviewer then asked the participant to recall the 16 common shopping list items again (delayed recall). The Spot-the-Word test (STW) was used in this study to test participants’ vocabulary and verbal knowledge with scores ranging from 0 to 60. STW testing involved presenting participants with pairs of items, one of which was a real word and the other a non-word, and then requiring participants to identify the word. Performance on the STW has not been shown to decline with age and is highly correlated with verbal acumen [25]. Finally, processing speed was tested using the Symbol-Digit Modalities Test (SDMT). Participants were provided with a reference key and asked to pair geometric figures with specific numbers. Using the SDMT, participants were scored from 0–60 on the number of correct answers provided in 90 s.

### 2.3. Food Consumption Data and Classification

The AusDiab semi-quantitative food frequency questionnaire consisted of 121 items that asked participants about their consumption of 101 food items [26]. This questionnaire assessed usual intake and recorded the amount and types of specific food items consumed by participants. In some cases, for example casseroles and potatoes, pictures of serving sizes were provided so that persons could indicate whether they had more or less of a given food item each day and each week, using the past 12 months as a reference. Participants were asked to specify the number of times they had specific food items in the past year by checking 1 of 10 frequency categories ranging from ‘never’ to ‘three or more times per day’. The average daily intake of food weight in grams was subsequently computed and used in the present analysis.

Three methods were used in order to classify these foods before applying PCA. In the first instance, the 101 individual food items asked about in the questionnaire were used (no categorisation). In the second and third instances, foods were combined and reduced to 32 and 20 food groups, respectively, based on nutrient content and culinary usage—a method employed in several other published studies for PCA [21,27] (see Table 1). Some foods were not categorised and were kept separate since they did not comfortably fit into any of the categories, e.g. pizza and meat pies [21,28]. More specifically, for the reduction of 101 items to 32 food groups, individual items were classed into groups, e.g., the item ‘Processed Meats’ was a tally of a participant’s bacon, ham, salami and sausage consumption in grams/day, while the item ‘Red Meats’ was a tally of beef, pork, lamb, veal and hamburger in grams/day. In the final classification system, the 32 food groups were further categorised into broader groups, which resulted in 20 food groups, e.g. the item ‘Meats’ was a tally of a participant’s ‘Processed Meats’ and ‘Red Meats’ consumption in grams/day.

**Table 1:** Food groupings used in the dietary pattern analysis.

*Method 1*	*Method 2*	*Method 3*
Food Item	Food Category	Food Category
Bacon, ham, salami, sausages	Processed Meats	Meat
Beef, pork, lamb, veal, hamburger	Red Meats	Meat
Fish, fried fish, tinned fish	Fish	Fish
Chicken	Poultry	Poultry
Eggs	Eggs	Eggs
Butter	Butter	Fats and Oils
Margarine, poly/mono-unsaturated margarine	Margarine	Fats and Oils
Butter and margarine blends	Butter and Margarine Blends	Fats and Oils
Reduced-fat/skim milk, low-fat cheese, yoghurt	Low-fat Dairy Products	Dairy
Full-cream milk, hard/firm/soft/ricotta/cottage/cream cheese, ice-cream, flavoured-milk drink	High-fat Dairy Products	Dairy
Red/white/fortified wine	Wine	Alcohol
Light/heavy beer	Beer	Alcohol
Other spirits	Other Spirits	Alcohol
Tinned fruit, oranges, apples, pears, bananas, melon, pineapple, strawberries, apricots, peaches, mango	Fruit	Fruit
Fruit juice	Fruit Juice	Fruit Juice
Cabbage, cauliflower, broccoli	Cruciferous Vegetables	Vegetables
Carrot, pumpkin	Dark-yellow Vegetables	Vegetables
Tomatoes, tomato sauce	Tomatoes	Vegetables
Lettuce, spinach	Green, leafy Vegetables	Vegetables
Peas, green beans, bean sprouts, baked beans, tofu, other beans, soya milk	Legumes	Vegetables
Cucumber, celery, beetroot, mushrooms, zucchini, capsicum, avocado	Other Vegetables	Vegetables
Onion, garlic	Garlic and Onions	Vegetables
Potatoes	Potatoes	Vegetables
Chips	Chips/French fries	Chips/French Fries
All-bran, bran flakes, Weet-Bix, cornflakes, porridge, muesli, wholemeal/rye/multi-grain bread	Whole Grains	Whole Grains
High-fibre white/white bread, rice, pasta, crackers	Refined Grains	Refined Grains
Pizza	Pizza	Pizza
Sweet biscuits, cakes, crisps, chocolate	Snacks	Snacks
Nuts, peanut butter	Nuts	Nuts
Jam, vegemite	Condiments	Condiments
Sugar	Sugar	Sugar
Meat pies	Meat Pies	Meat Pies

### 2.4. Statistical Analysis

PCA using SPSS version 22 was conducted to identify underlying dietary patterns. In determining the number of components to retain for further analysis, we considered component eigenvalues greater than 1 along with examination of scree plots. Components were rotated by an orthogonal (varimax) rotation to improve interpretability. Overall though, the comprehensibility and interpretability of the rotated factors were considered along with the aforementioned criteria. Similar to other studies, derived components were labelled based on our description of the observed patterns [29].

Dietary pattern scores were calculated for each individual at Wave 1 using all three classification methods (individual food items, 32 food groups and 20 food groups). Scores for an observed pattern were computed using the following equation: $i=\;\sum_{j}\left[\left(b_{ij}/\lambda_{i}\right)X_{j}\right]\;$ [29]. Variables with factor loadings of ≥0.30 were included in the weighted average [30,31].

Logistic regression analysis was performed to determine the association between dietary pattern scores at Wave 1 and cognitive status at Wave 3 using all three food item categorisation methods, *i.e.* PCA based on 101 individual food items, 32 food groups or 20 food groups. The interaction between dietary pattern score and exercise time was also examined to determine whether there was any association with cognitive status using all three food categorisation methods.

Generalized linear models (GLM) were used to estimate the associations between dietary pattern scores at Wave 1 and memory, verbal ability and processing speed using all three food variable reduction methods.

## 3. Results

Descriptive statistics for the sample are presented in Table 2. A total of 577 participants (49.22% female) had both diet and cognitive data recorded at Wave 1.

**Table 2:** Descriptive statistics at Wave 1 for the AusDiab sample included in the study (*n* = 577).

Variables	Wave 1
Age Range	60–83
Mean Age (SD)	66.07 (4.85)
Female (%)	284 (49.22)
BMI (SD)	26.89 (4.09)
Secondary School (%)	242 (24.4)
Tertiary Level (%)	229 (40.1)
Other - Trade, Technician, Primary Only (%)	100 (17.4)
Current Smoker (%)	29 (5.1)
Ex-Smoker (%)	182 (32.0)
Non-Smoker	357 (62.9)
Exercise Mean (SD), mins./week	292.45 (324.21)
MMSE Score	27.41 (2.44)
CVLT Score	5.17 (2.30)
STW Score	50.30 (6.84)
SDMT Score	38.63 (10.74)
Impaired (%)	44 (7.63)

### 3.1. Dietary Pattern Analysis

Classification method affected the number and components of the patterns identified. Variable reduction using 20 food groups explained a greater proportion of variance in the sample than variable reduction using 32 food groups and 101 individual food items. Use of 20 food groups explained 30.74% of total variance in food use in the sample. Comparatively, use of 101 individual foods explained 23.22% and use of 32 food groups explained 29.74% of total variance in food use.

**Table 3:** Results of logistic regression analyses showing associations between CI at Wave 3 and dietary patterns obtained using 101 food items, 32 food groups and 20 food groups at Wave 1 (odds ratios with 95% confidence intervals shown in brackets).

	Dietary Pattern 1	Dietary Pattern 2	Dietary Pattern 3	Dietary Pattern 4	Dietary Pattern 5	Dietary Pattern 6	Dietary Pattern 7
101 Food Items	Fruit & Vegetable	Snack & Processed Foods	Vegetable	Meat	Fish, Legumes & Vegetable	Vegetable, Pasta & Alcohol	Dairy, Cereal & Eggs
OR (95% CI)	1.061 (1.006–1.118) *p* = 0.030 *	1.051 (0.967–1.143) *p* = 0.239	0.986 (0.916–1.061) *p* = 0.701	1.005 (0.964–1.048) *p* = 0.806	1.032 (1.001–1.064) *p* = 0.040 *	1.000 (0.965–1.037) *p* = 0.994	1.020 (1.007–1.033) *p* = 0.003 **
32 Food Groups	Western	Prudent	Vegetable, Grains & Wine	High-Fat			
OR (95% CI)	1.005 (0.994–1.016) *p* = 0.409	0.997 (0.984–1.010) *p* = 0.643	1.008 (0.995–1.020) *p* = 0.229	0.999 (0.992–1.007)			
20 Food Groups	Variety	Western	Dairy, Grains & Alcohol				
OR (95% CI)	1.006 (0.994–1.018) *p* = 0.333	1.008 (0.986–1.031) *p* = 0.497	1.001 (0.998–1.005) *p* = 0.383				

Model adjusted for age, sex, energy, education, BMI, smoking status, STW and exercise time; * *p* < 0.05; ** *p* < 0.01.

#### 3.1.1. Wave 1 Dietary Patterns Using 101 Individual Food Items

Seven dietary patterns were extracted using PCA with varimax rotation. The rotated component matrix with factor loadings is shown in Supplementary Table 1.

The first dietary pattern identified was labelled ‘Fruit and Vegetable’ because of the high loadings of unprocessed fruit and vegetables observed. The second dietary pattern identified was labelled ‘Snack and Processed Food’ due to the high factor loadings observed for foods that could be qualified as such, e.g., cakes, jam, ice cream, sausages, and salami. Dietary pattern labels for the other observed dietary structures can be viewed in Supplementary Table 1. Together, the dietary patterns identified accounted for 23% of total variance in the sample.

#### 3.1.2. Wave 1 Dietary Patterns Using 32 Food Groups

After applying PCA, four dietary patterns were extracted. The rotated component matrix with factor loadings is shown in Supplementary Table 2.

The first pattern identified was labelled ‘Western’ because of the predominantly high loadings of processed meats, refined grains and convenience foods. The second dietary pattern identified was labelled ‘Prudent’ and had characteristically high factor loadings of fish, vegetables and fruit. Dietary pattern labels for the other observed dietary structures can be viewed in Supplementary Table 4. Together, the dietary patterns identified accounted for 30% of total variance in the sample.

#### 3.1.3. Wave 1 Dietary Patterns Using 20 Food Groups

Three dietary patterns were extracted using PCA with varimax rotation. The rotated component matrix with factor loadings is shown in Supplementary Table 3.

The first pattern identified was labelled ‘Variety’ because of the high loadings of a wide variety of foods—vegetables, fruit, fish, meat and nuts. The second dietary pattern was labelled ‘Western’ because of the high factor loadings of high-fat and high-sugar foods. The final dietary pattern was labelled ‘Dairy, Grains and Alcohol’ due to the high factor loadings of these foods recorded. Together, these dietary patterns identified accounted for 31% of total variance in the sample.

### 3.2. Dietary Pattern as a Predictor of CI Using the MMSE

Logistic regression analysis using dietary pattern scores obtained from all three variable reduction techniques (*i.e.*, 101 individual food items, 32 food groups and 20 food groups) was conducted to examine the relationship between dietary pattern and CI. Covariates included the independent variables age, sex, energy, education, BMI, smoking status, exercise time and Spot-the-Word (as a control for premorbid intelligence) [32].

The only significant dietary predictors of CI were obtained using 101 individual food items. Three of the seven dietary patterns identified were observed to be significant predictors of CI. For every one unit increase in these pattern scores, the odds of CI decreased ((Fruit and Vegetable Pattern: *p* = 0.030, OR 1.061, confidence interval: 1.006–1.118); (Fish, Legumes and Vegetable Pattern: *p* = 0.040, OR 1.032, confidence interval: 1.001–1.064); (Dairy, Cereal and Eggs Pattern: *p* = 0.003, OR 1.020, confidence interval: 1.007–1.033)).

**Table 4:** Results of GLM showing associations between cognitive function at Wave 3 and dietary patterns obtained using 101 individual food items, 32 food groups and 20 food groups at Waves 1, 2 and 3 (β values with Standard Errors shown in brackets).

	Dietary Pattern 1	Dietary Pattern 2	Dietary Pattern 3	Dietary Pattern 4	Dietary Pattern 5	Dietary Pattern 6	Dietary Pattern 7
101 Food Items	Fruit & Vegetable	Snack & Processed Foods	Vegetable	Meat	Fish, Legumes & Vegetable	Vegetable, Pasta & Alcohol	Dairy, Cereal & Eggs
CVLT	0.012 (0.013) *p* = 0.336	0.020 (0.015) *p* = 0.186	−0.001 (0.012) *p* = 0.930	0.000 (0.005) *p* = 0.984	−0.002 (0.007) *p =* 0.793	0.004 (0.006) *p =* 0.551	−4.474 (0.002) *p =* 0.986
SDMT	0.097 (0.057) *p =* 0.091	0.060 (0.067) *p =* 0.365	0.013 (0.054) *p =* 0.801	0.014 (0.023) *p =* 0.536	−0.062 (0.032) *p =* 0.054	−0.003 (0.028) *p =* 0.916	−0.016 (0.011) *p =* 0.149
STW	0.077 (0.039) *p =* 0.051	0.080 (0.046) *p =* 0.086	0.046 (0.037) *p =* 0.224	0.007 (0.020) *p =* 0.722	0.000 (0.022) *p =* 0.994	0.000 (0.021) *p =* 0.982	0.002 (0.008) *p =* 0.799
32 Food Groups	Western	Prudent	Vegetable, Grains & Wine	High-Fat			
CVLT	−0.008 (0.003) *p =* 0.001 **	−0.005 (0.003) *p =* 0.067	0.001 (0.003) *p =* 0.764	−0.001 (0.001) *p =* 0.711			
SDMT	−0.024 (0.011) *p =* 0.035 *	−0.035 (0.011) *p =* 0.002 **	0.024 (0.012) *p =* 0.034 *	0.005 (0.006) *p =* 0.403			
STW	−0.006 (0.008) *p =* 0.467	−0.006 (0.008) *p =* 0.425	0.013 (0.008) *p =* 0.119	0.001 (0.005) *p =* 0.774			
20 Food Groups	Variety	Western	Dairy, Grains & Alcohol				
CVLT	−0.003 (0.003) *p =* 0.272	−0.004 (0.005) *p =* 0.376	−0.002 (0.001) *p =* 0.005**				
SDMT	−0.026 (0.011) *p =* 0.018 *	−0.007 (0.021) *p =* 0.740	−0.005 (0.003) *P =* 0.149				
STW	−0.008 (0.008) *p =* 0.291	0.002 (0.015) *p =* 0.901	−0.001 (0.002) *p =* 0.618				

Model adjusted for age, sex, energy, education, BMI, smoking status, STW and exercise time; * *p* < 0.05, ** *p* < 0.01.

When the interaction term ‘dietary pattern score × exercise time’ was included in the model, no significant results were obtained.

### 3.3. Dietary Pattern as a Predictor of Memory, Vocabulary and Verbal Knowledge and Processing Speed

Using dietary pattern scores calculated from 101 individual food items, there were no dietary patterns observed to be significantly predictive of memory, processing speed or verbal knowledge.

Using 32 food groups, however, the ‘Western’ dietary pattern was a predictor of poorer memory and processing speed (β = −0.008, SE = 0.003, *p* = 0.001 and β = −0.024, SE = 0.011, *p* = 0.035). In addition, the ‘Prudent’ dietary pattern was also a predictor of poorer processing speed (β = −0.035, SE = 0.011, *p* = 0.002). 

When 20 food groups were used to calculate dietary pattern scores, the ‘Dairy, grains and alcohol’ dietary pattern was predictive of poorer memory while the ‘Variety’ dietary pattern was associated with poorer processing speed ( = −0.002, SE = 0.001, *p* = 0.005 and When 20 food groups were used to calculate dietary pattern scores, the ‘Dairy, grains and alcohol’ dietary pattern was predictive of poorer memory while the ‘Variety’ dietary pattern was associated with poorer processing speed (β = −0.002, SE = 0.001, *p* = 0.005 and β = −0.026, SE = 0.011, *p* = 0.018 respectively).

## 4. Discussion

The present study is one of the few that use a data-driven method of dietary analysis to assess the relationship between diet and cognitive function in older Australian adults. A number of findings from this study are noteworthy. First, the broader the categories used in grouping foods, the greater the variability in food use that was explained. It was observed, however, that the results of logistic regression were more sensitive when dietary analysis was based on individual food items than food groups. From these data we observed that for every one unit increase in ‘Fruit and Vegetable’, ‘Fish, Legumes and Vegetable’ and ‘Dairy, Cereal and Eggs’ dietary pattern scores, the odds of CI decreased.

When looking at the relationship between dietary pattern and memory, processing speed and vocabulary, no significant results were observed using 101 individual food items. Using 32 food groups, the ‘Western’ dietary pattern was found to be predictive of poorer memory and processing speed, the ‘Vegetable, Grains and Wine’ pattern was a predictor of better processing speed while the ‘Prudent’ pattern was predictive of poorer processing speed. Using 20 food groups we observed that the ‘Variety’ dietary pattern was a predictor of poorer processing speed and the ‘Dairy, Grains and Alcohol’ pattern predictive of poorer memory.

We found that the method of reducing food variables affected the amount of variance in food use that was explained. Similarly to other published findings, the broader the categories used, the greater the variability in food use explained [21]. We suggest this may be due to the inclusion of foods that are both weakly and strongly correlated with a specific pattern in the broader categorisations which leads to an increase in the information captured [21]. Interestingly, for logistic regression analyses, it was only when the level of detail in the items included in PCA-derived dietary patterns increased that significant associations between diet and cognitive impairment were observed, *i.e.*, it was variable reduction that utilised dietary pattern scores from individual foods that produced the only significant results. This may be because analysis using individual foods captures more meaningful results as it is able to show whether consumption or non-consumption of specific food items is associated with disease.

Our results are consistent with previous studies in showing that diets with high loadings of vegetables and other plant-based food items (fruit, grains and legumes) resulted in reduced odds of disease and improved cognitive function [21,27]. In a study of 6911 Chinese subjects aged 65 and older who formed part of the Chinese Longitudinal Health Longevity Study, lower intakes of vegetables and legumes were associated with cognitive decline when using MMSE as a measure of cognitive function [33]. Multivariate logistic regression showed that always eating vegetables and always consuming legumes were inversely associated with cognitive decline [33]. Additionally, in a study of 2,148 community-based elderly subjects without dementia in New York, higher intakes of cruciferous and dark and green leafy vegetables were found to be associated with a decreased risk of developing AD [34]. The benefits of diets high in vegetables extend beyond the cognitive domain. In a European study aimed to investigate the effect of the Mediterranean diet (MeDi) on mortality, greater adherence was associated with a more than 50% lower rate of all-causes and cause-specific mortality [35]. McCann *et al*. [21], in a study examining the effect of dietary patterns on estimation of endometrial cancer risk, found that dietary patterns high in fruit, vegetables and whole-grains resulted in reduced endometrial cancer risks. Similarly, dietary patterns high in vegetables, grain and fruit have been found to be associated with a modestly lower risk for type 2 diabetes [27]. This seemingly protective association between plant-based foods and disease may be the result of the high concentration of antioxidant nutrients present in vegetables and fruits and their role in suppressing inflammation. There is evidence that oxidative stress and inflammation can lead to impaired cognitive function because of an increase in free radicals and the damage they cause to neuronal cells [14].

The Mediterranean diet (MeDi), one of the most studied dietary patterns, describes a diet rich in cereals, olive oil, fish, fruits and vegetables and low in dairy and meat with a moderate consumption of red wine. This diet has been linked to increased survival, reduced risk of cancers, cardiovascular disease, longevity and CI [11,16,36,37,38]. In the present study, diets rich in vegetables, grain and wine were found to be predictive of better processing speed and diets high in vegetables and plant-based food items were generally associated with better cognitive outcomes.

Worth noting is the finding that the ‘Prudent’ dietary pattern was predictive of poorer processing speed. This dietary pattern was so labelled because of its high loadings of fish, fruit and vegetables, nuts and whole-grains. Perhaps an explanation of this lies in the method in which food items are prepared or in analysing whether there is actually a protective effect of foods contained in this pattern. For instance, while many studies have examined the effect of fish consumption on cognition, some clarification is still needed on the purported link between the two. In a study of 6150 Chicago residents aged 65 and older to examine whether intake of fish and omega-3 fatty acids protects against age-related cognitive decline, it was reported that fish consumption may be associated with slower cognitive decline with age [39]. Similar findings were also reported by Kalmijn *et al*. in 1997, who found statistically significant decreased risks of AD with higher fish consumption [40]. In two more recent Australian studies, one reported that higher fish consumption was associated with an increased risk of cognitive disorder [15] while the other found no evidence to support the hypothesis that higher proportions of fish intake benefits cognitive performance in normal older adults [41].

The ‘Variety’ dietary pattern was also found to be predictive of poorer processing speed. This pattern, so named because of high factor loadings in a variety of foods, is of interest because dietary guidelines for Australia and the rest of the world highlight the benefits of consuming a wide variety of foods. Therefore, in food and nutrition policy, there is a need to ensure that messages about the method of food preparation, processing and portion sizes of consumables are equally stressed.

In the present study, the ‘Western’ dietary pattern was predictive of poorer memory and processing speed. This is supported by other research which has reported that the ‘Western’ dietary pattern is associated with cognitive decline and reduced executive function [42].

One of the limitations of this study lies in its inability to report disease incidence as cognitive data were only collected at one time point (Wave 3). Additionally, no data were collected on executive function, and dietary intake is self-reported. There is also some subjectivity in determining food groups before application of PCA, but the method of food variable reduction we employed has been widely used in other studies [21,27]. It is also possible that the results observed may represent a selection bias, as only older adults with dietary and cognitive data were included in the study (*n* = 577). This has the effect of limiting the generalizability of the study’s findings. Finally, while we focused on the methodology of grouping foods in this paper, we were still unable to clearly identify guidelines for future researchers to follow. This is a major issue for diet-cognition research and suggests the need for further investigation and development of more robust and consensus-led methodologies in the field.

Despite its limitations, this study adds to the sparse body of literature examining the relationship between dietary patterns and CI among older adults, both in Australia and internationally. Furthermore, the study’s focus on older age groups whose dietary patterns have not been widely studied and reported is noteworthy. Finally, the study’s ability to answer a methodological question that has been one of the main critiques of PCA makes it noteworthy—how do variable reduction methods before the application of PCA affect the results obtained? This question is significant when examining the relationship between dietary patterns and cognition since there is a level of subjectivity involved in reducing food variables, and these can affect the observed associations with cognitive function [27].

Future studies examining the association between dietary intake and cognitive status will be useful to identify other patterns associated with CI and to examine more nuanced issues as they relate to diet and cognitive function.

## 5. Conclusions

Our findings showed that diets with high factor loadings of fruit, vegetables and plant-based food items conferred cognitive benefits, while those with high factor loadings of high-fat and convenience foods are linked to poorer cognitive outcomes. These results are similar to those of other studies which show that diets with high loadings of vegetables, fruit and grain reduce the odds of a myriad of diseases [21,27,43]. In addition, we demonstrated that the method of variable reduction in dietary studies may influence results, and suggest that further work is required to establish robust and replicable methods of dietary analysis for use in research into cognitive ageing. Additional studies that focus on the dietary habits of those over age 60 would be useful in order to further elucidate more specific details between dietary patterns, types and amount of fat, protein and carbohydrates, number of calories, and micro and macronutrients that are linked with optimal cognitive function and reduced risk of CI in older adults. Such information is required to provide support for the development of policies that promote optimal cognitive health in ageing.

## Supplementary Files

Supplementary File 1
