# Supplementary material for: Role of Dietary Pattern Analysis in Determining Cognitive Status in Elderly Australian Adults
Source: Nutrients. 2015 Feb 4;7(2):1052–67. doi: 10.3390/nu7021052 (PMC4344574; doi:10.3390/nu7021052)
Supplement: Supplementary File 1 [file nutrients-07-01052-s001.docx]

**Supplementary Information**

**Table S1.** Factor—loading matrix for 7 major dietary patterns identified at Wave 1 using 101 individual food items.

| **Food Item** | **Pattern 1 (Fruit and Vegetable)** | **Pattern 2  (Snack & Processed Food)** | **Pattern 3 (Vegetable)** | **Pattern 4 (Meat)** | **Pattern 5 (Fish & Plant-based)** | **Pattern 6 (Veg, Pasta, Alcohol)** | **Pattern 7 (Dairy, Cereals & Eggs)** |
| --- | --- | --- | --- | --- | --- | --- | --- |
| Carrots | 0.705 |  |  |  |  |  |  |
| Onion | 0.563 |  |  |  |  |  |  |
| Beetroot | 0.533 |  |  |  |  |  |  |
| Pumpkin | 0.507 |  |  |  |  | −0.373 |  |
| Mango | 0.465 |  |  |  |  |  |  |
| Zucchini | 0.452 |  |  |  |  |  |  |
| Avocado | 0.435 |  | 0.348 |  |  |  |  |
| Peaches | 0.430 |  |  |  |  |  |  |
| Apricots | 0.404 |  |  |  |  |  |  |
| Crackers | 0.376 |  |  |  |  |  | 0.331 |
| Potatoes | 0.371 | 0.313 |  |  |  |  |  |
| Multigrain bread | 0.337 |  |  |  |  |  |  |
| Cakes |  | 0.595 |  |  |  |  |  |
| Jam |  | 0.524 |  |  |  |  |  |
| Ice cream |  | 0.522 |  |  |  |  |  |
| Sweet Biscuits |  | 0.462 |  |  |  |  |  |
| Salami |  | 0.447 |  |  |  |  |  |
| Sausages |  | 0.394 |  |  |  |  |  |
| Chocolate |  | 0.387 |  |  |  |  |  |
| Sugar |  | 0.370 | −0.319 |  |  |  |  |
| Meat pies |  | 0.356 |  |  |  |  |  |
| Vegemite |  | 0.317 |  |  |  |  |  |
| Lettuce |  |  | 0.580 |  |  |  |  |

**Table S1.** *Cont.*

| Cucumber |  |  | 0.566 |  |  |  |  |
| --- | --- | --- | --- | --- | --- | --- | --- |
| Capsicum |  |  | 0.434 |  |  |  |  |
| Peas | 0.310 |  | −0.426 |  |  |  |  |
| Celery |  |  | 0.415 |  |  |  |  |
| Tomatoes |  |  | 0.398 |  |  |  |  |
| Chips |  |  | −0.361 |  |  |  |  |
| Yoghurt |  |  | 0.357 |  |  |  |  |
| White bread |  |  | −0.327 |  |  |  |  |
| Pork |  |  |  | 0.782 |  |  |  |
| Veal |  |  |  | 0.757 |  |  |  |
| Lamb |  |  |  | 0.705 |  |  |  |
| Beef |  |  |  | 0.628 |  |  |  |
| Chicken |  |  |  | 0.426 |  |  |  |
| Peanut butter |  |  |  | 0.331 |  |  |  |
| Soft cheese |  |  |  | 0.306 |  |  |  |
| Fish |  |  |  |  | 0.520 |  |  |
| Other beans |  |  |  |  | 0.505 |  |  |
| Tofu |  |  |  |  | 0.505 |  |  |
| Spinach |  |  |  |  | 0.463 |  |  |
| Baked beans |  |  |  |  | 0.408 |  |  |
| Tinned fish |  |  |  |  | 0.390 |  |  |
| Ricotta or cottage cheese |  |  |  |  | 0.384 |  |  |
| Mushrooms | 0.312 |  |  |  | 0.380 |  |  |
| Bean sprouts |  |  |  |  | 0.363 |  |  |
| Strawberries |  |  |  |  | 0.354 |  |  |
| Fried fish |  |  |  |  | 0.319 |  |  |
| Soya milk |  |  |  |  | 0.301 |  |  |
| Cauliflower |  |  |  |  |  | −0.519 |  |

**Table S1.** *Cont.*

| Broccoli | 0.304 |  |  |  |  | −0.415 |  |
| --- | --- | --- | --- | --- | --- | --- | --- |
| Cabbage | 0.366 |  |  |  |  | −0.372 |  |
| Red wine |  |  |  |  |  | 0.363 |  |
| Pasta |  |  |  |  |  | 0.347 |  |
| Spirits |  |  |  |  |  | 0.301 |  |
| Full cream milk |  |  |  |  |  |  | −0.478 |
| Low-fat cheese |  |  |  |  |  |  | 0.448 |
| Tinned fruit |  |  |  |  |  |  | 0.440 |
| Cornflakes |  |  |  |  |  | 0.331 | 0.369 |
| Fruit juice |  |  |  |  |  |  | 0.368 |
| Eggs |  |  |  |  |  |  | −0.356 |
| All bran |  |  |  |  |  |  | 0.323 |
| Butter and margarine blends |  |  |  |  |  |  | −0.317 |

**Table S2.** Factor—loading matrix for 4 major dietary patterns identified at Wave 1 using 32 Food Groups.

| **Food Group** | **Pattern 1 (Western)** | **Pattern 2 (Prudent)** | **Pattern 3 (Veg., Grains & Wine)** | **Pattern 4 (High fat)** |
| --- | --- | --- | --- | --- |
| Processed Meats | 0.640 |  |  |  |
| Red Meats | 0.554 |  |  |  |
| Snacks | 0.507 |  | 0.375 |  |
| Refined Grains | 0.432 |  |  |  |
| Poultry | 0.404 |  |  |  |
| Condiments | 0.392 |  | 0.377 |  |
| Meat Pies | 0.377 |  |  |  |
| Chips/French Fries | 0.367 |  |  |  |
| Beer | 0.356 |  |  |  |
| Pizza | 0.315 |  |  |  |
| Other Vegetables |  | 0.704 |  |  |
| Green Leafy Vegetables |  | 0.544 |  |  |
| Fruit |  | 0.540 |  |  |
| Garlic and Onions |  | 0.518 |  |  |
| Fish |  | 0.429 |  |  |
| Nuts |  | 0.343 |  |  |
| Tomatoes |  | 0.335 |  |  |
| Dark Yellow Vegetables |  |  | 0.732 |  |
| Potatoes |  |  | 0.660 |  |
| Cruciferous Vegetables |  |  | 0.519 |  |
| Whole Grains |  |  | 0.395 |  |
| Wine |  |  | −0.336 |  |
| High Fat Dairy Products |  |  |  | 0.689 |
| Low Fat Dairy Products |  |  |  | −0.641 |
| Margarine |  |  |  | −0.593 |
| Butter |  |  |  | 0.510 |
| Butter and Margarine Blends |  |  |  | 0.441 |
| Eggs |  |  |  | 0.349 |

**Table S3.** Factor—loading matrix for 3 major dietary patterns identified at Wave 1 using 20 Food Groups.

| **Food Group** | **Pattern 1 (Variety)** | **Pattern 2 (Western)** | **Pattern 3 (Dairy, Grains & Alcohol)** |
| --- | --- | --- | --- |
| Vegetables | 0.566 |  |  |
| Fish | 0.549 |  |  |
| Fruit | 0.544 |  |  |
| Poultry | 0.543 |  |  |
| Nuts | 0.497 |  |  |
| Condiments | 0.459 | 0.371 |  |
| Meat | 0.418 |  | 0.393 |
| Fruit Juice | 0.392 |  |  |
| Sugar |  | 0.580 |  |
| Snacks |  | 0.545 |  |
| Fats and Oils |  | 0.530 |  |
| Meat Pies |  | 0.474 |  |
| Chips/French Fries |  | 0.448 |  |
| Legumes |  | −0.378 |  |
| Dairy |  |  | −0.611 |
| Whole Grains | 0.420 |  | −0.564 |
| Alcohol |  |  | 0.516 |
| Refined Grains |  |  | 0.393 |

© 2015 by the authors; licensee MDPI, Basel, Switzerland. This article is an open access article distributed under the terms and conditions of the Creative Commons Attribution license (http://creativecommons.org/licenses/by/4.0/).
